# Custom-made 3D-printed cup-cage implants for complex acetabular revisions: evaluation of pre-planned versus achieved positioning and 1-year migration data in 10 patients

**DOI:** 10.1080/17453674.2020.1819729

**Published:** 2020-09-15

**Authors:** Vasileios Zampelis, Gunnar Flivik

**Affiliations:** Department of Orthopedics, Skane University Hospital, Clinical Sciences, Lund University, Lund, Sweden

## Abstract

Background and purpose — The use of custom-made 3D-printed titanium implants for the reconstruction of large acetabular defects has been successively introduced in the last decade. In an observational cohort study we evaluated the agreement between preoperatively planned and actually achieved cup-cage position as well as 1-year follow-up migration of the cup-cage component.

Patients and methods — 10 patients with Paprosky III defects underwent revision surgery using a custom-made 3D-printed cup-cage. The position of the implant on postoperative CT scan was compared with the preoperative plan and the postoperative CT scan was compared with the 1-year follow-up CT scan.

Results — There was a median deviation in postoperative position versus planned in inclination of 3.6° (IQR 1.0–5.4), in anteversion of –2.8° (IQR –7.5 to 1.2), and in rotation of –1.2° (IQR –3.3 to 0.0). The median deviation in position of the center of rotation (COR) was –0.5 mm (IQR 2.9 to 0.7) in the anteroposterior (AP) plane, –0.6 mm (IQR –1.8 to –0.1) in the mediolateral (ML) plane, and 1.1 mm (IQR –1.6 to 2.8) in the superoinferior (SI) plane. The migration between postoperative and 1-year follow-up caused a mean change in inclination of 0.04° (IQR –0.06 to 0.09), in anteversion of –0.13° (IQR –0.23 to –0.06), and in rotation of 0.05° (IQR –0.46 to 1.4). The migration of COR was –0.08 mm (IQR –0.18 to –0.04) in the AP plane, 0.14 mm (IQR –0.08 to 0.22) in the ML plane, and 0.06 mm (IQR –0.02 to 0.35) in the SI plane. There was no re-revision.

Interpretation — The early results show good agreement between planned and achieved cup-cage position and small measured migration values of the cup-cage component at the 1-year follow-up.

Revision of the acetabular component following primary total hip arthroplasty (THA) is especially challenging in patients with large bony defects. The Paprosky classification (Paprosky et al. 1994) is a widely used system for classifying acetabular bone loss in revision THA. The Paprosky III defects are the most complex patterns and therefore the most difficult to reconstruct.

The aim of acetabular revision is to restore, when possible, the bone defects present and the center of rotation (COR) of the hip joint, thus providing a stable and durable reconstruction. Various reconstructive surgical techniques exist depending on whether the bone defects are small/contained or larger/non-contained.

In our department, for the treatment of such large acetabular bone defects, we have started to use the aMace acetabular revision system (aMace Cage, Materialise, Leuven, Belgium), a custom-made titanium cup-cage implant. The implant is designed from a computed tomography (CT) analysis of the bone-deficient acetabulum, providing a 3D-printed titanium cup-cage into which to cement a cup. The implant used was designed without an integrated augment but to fully match the individual patient’s outer pelvic surface anatomy, thus resulting in a stable reconstruction and bridging of larger bone defects. There are locations for pre-planned multiple screws, to fix the implant, aiming for the areas with best bone quality. The personalized design is intended to maximize bone preservation, allowing adaptation of the 1-piece implant to the specific patient in which a cemented cup can be placed in the desired position, thus restoring the hip COR.

We evaluated the agreement between planned and achieved cup-cage position and measured possible migration of the cup-cage component, by using a novel CT-measuring technique, in patients with Paprosky III acetabular defects at 1-year follow-up.

## Patients and methods

### Study design

This is a single-center observational cohort study. The inclusion criteria were patients, regardless of age, with aseptic prosthetic loosening following either a primary total hip arthroplasty (THA) or 1 or multiple earlier revisions, with a Paprosky type III-A acetabular bone defect (Table 1). The patients were scheduled for acetabular revision surgery with a custom-made 3D-printed cup-cage (aMace Cage, Materialise, Leuven, Belgium) revision system and surgery was performed between March 2014 and May 2017. Due to large bone defects present in these patients, few other surgical options were available.

**Table 1.** Patient demographics. Values are number unless otherwise specified

Variable	
No. of patients	10
Mean age (range)	64 (36–87)
Sex (M:F)	5:5
Mean BMI (range)	26 (19–34)
Paprosky III-A	10
Right:left	7:3
First-time vs. repeat revisions	3:7

### Cup-cage design

**Figure 1.**
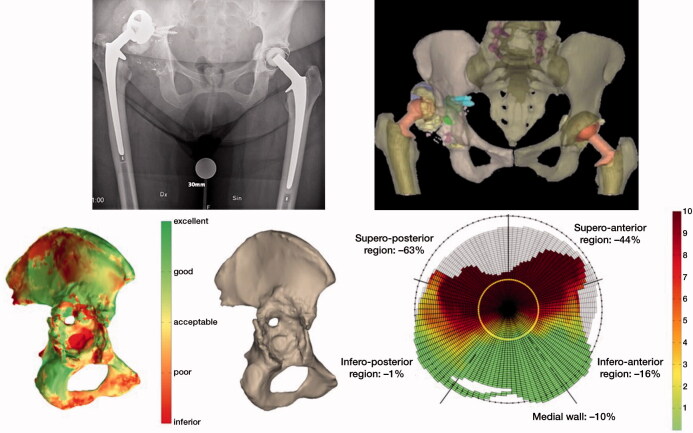
Bone quality assessed by the software; red color reveals a poor and green shows excellent bone quality. (a): Assessment of bone quality as described by Gelaude. (b): Defect analysis quantifies in percentages and colors bone loss in the different regions of the acetabulum on which the Paprosky classification is based. Red color reveals an inferior and green shows excellent bone quality while yellow is acceptable. The output data consists of a ratio and a graph, which allow the direct comparison between specimens. The amount of original acetabular bone that is missing is defined as a ratio. The remaining bone stock in the radial direction is presented by the graph (c).

**Figure 2.**
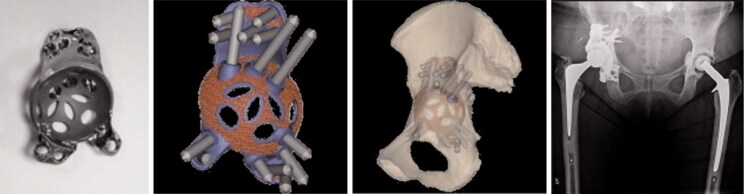
Custom made impalnt, planned screw positions and implant position based on CT scan. CT was taken prior to surgery. The radiograph shows the postoperative result. The position of the implant after surgery was compared with the preoperative planning using the pre- and postoperative CT scans.

Each patient had a routine preoperative CT scan to determine the acetabular defects in terms of missing bone and thickness, based on which the Paprosky type was determined (Figure 1). Based on the preoperative CT scan, the implant was designed to achieve the ideal center of rotation (COR), optimal implant inclination (INCL), and anteversion (AV). Additionally, a unique bone quality map was provided to determine the total radial acetabular bone loss (Gelaude et al. 2011) (Figure 1) and the ideal screw positioning. Thus, the CT scan data was used for the production of a 3D-printed bone model of the patient’s hemipelvis, an implant trial model, drill guides, and finally a patient-specific monobloc cup-cage titanium implant (Figure 2). The implants we used had no integrated augments but a porous rough trabecular back surface designed to improve secondary fixation and central holes in the dome to be able to impact bone graft.

### Participants

10 patients (mean age 64 years [36–87], 5 men) with Paprosky III-A acetabular defects scheduled for acetabular cup revision with the use of a custom-made 3D-printed cup-cage (aMace Cage, Materialise, Leuven, Belgium) were included (Table 1). All patients remained available for the follow-up CT scan 1 year after surgery. Revision involved both cup and stem components.

### Surgery

All revisions were carried out at the Orthopedic Department of Skåne University Hospital (Lund University, Sweden) and performed through a posterolateral approach by the same orthopedic surgeon (GF). After removal of the failed implant, all interface membranes in the acetabulum and areas of bone defect were removed, exposing the underlying acetabular morphologic features as modelled during prototyping. In all cases, prior to cage placement, morselized allograft bone was used to fill the gap between the cage and the host bone. Information regarding the quantity of allograft bone used in the impaction bone grafting was not recorded in all cases. In those cases the quantity was recorded; 1 large or 2 medium femoral heads were used.

The custom-made implant was fixated with screws, the number, positions, and length of which were according to the preoperative plan based on the CT scan. An Exeter X3 RimFit cup (Stryker International, London, UK) was cemented with the same AV and INCL as the cup-cage implant itself. After surgery, while the patient was still in hospital, a postoperative CT was performed. Postoperative full weight-bearing was allowed, as tolerated.

3 doses of systemic cloxacillin were given as perioperative prophylaxis. Low molecular weight heparin was given postoperatively for 10 days but 4 weeks if predisposed with any extra thromboembolic risk factors.  

### CT analysis and evaluation

For each patient, 3 CT scans were performed: 1 prior to surgery, 1 during the 1st postoperative week (CT1), and the final at 1-year follow-up (CT2). 30 CT scan sets were thus included in this analysis. For every patient, each postoperative CT examination was analyzed twice (M1 and M2), at least 1 month apart. The rater (at Materialise) was blinded to the 1st set of results by renaming the case IDs in the filename of the image sets to avoid potential bias. Repeatability, assuming no bias (M1, M2), was calculated as described by Ranstam et al. (2000) (Table 2).

**Table 2.** Median difference between planned versus postoperative plus migration values from direct postoperative to 1-year follow-up

Factor	Deviation from planned cup position			First year migration			Repeatability limit **^a^**	
	median	IQR	range	median	IQR	range	Postop.	1 year
Inclination (°)	3.6	1.0 to 5.4	–11 to 11	0.04	–0.06 to 0.09	–0.22 to 0.98	0.48	0.51
Anteversion (°)	–2.8	–7.5 to 1.2	–12 to 5.7	–0.13	–0.23 to –0.06	–0.41 to 0.05	2.1	1.0
Rotation (°)	–1.2	–3.3 to 0.0	–7.4 to 14	0.05	–0.07 to 0.36	–0.46 to 1.4	0.97	0.99
Translation of COR (mm)								
AP plane (anterior +)	–0.5	–2.9 to 0.7	–7.0 to 3.4	–0.08	–0.18 to –0.04	–0.48 to 0.43	0.28	0.25
ML plane (medial +)	–0.6	–1.8 to –0.1	–3.6 to 2.2	0.14	–0.08 to 0.22	–0.35 to 0.51	0.63	0.34
SI plane (superior +)	1.1	–1.6 to 2.8	–9.7 to 8.9	0.06	–0.02 to 0.35	–0.15 to 0.61	0.44	0.26

Values are presented as signed, thus indicating the direction of the migration except for rotation where signed values are positive for clockwise rotation after signed values for left hips are shifted.

**^a^** Repeatability limit: the value less than or equal to which the absolute difference between two tests results obtained under repeatability conditions may be expected to have a probability of 95%.

IQR: interquartile range.

For deviation analysis the achieved position of the implant on postoperative CT scan (CT1) was measured and compared with the preoperatively planned position, using the average values of the 2 repeated measures (M1, M2). Further, the position on the 1-year follow-up CT scan was also compared with postoperative CT scan. For the migration analysis, the difference between these 2 measurements was calculated. All CT scans were analyzed by the same software from Materialise (Mimics Innovation Suite, Materialise NV, Leuven, Belgium).

As the same orthogonal coordinate system is used for the measurements of both left and right hips, to make them comparable, the signed values were shifted for the left hips regarding inclination, anteversion and mediolateral translation. Inclination, anteversion and the center of rotation (COR) of the implant were then compared using signed values, indicating the actual direction of the changes in positioning and migration. The position of the COR was decomposed into 3 different orthogonal components: anteroposterior (AP), mediolateral (ML), and superoinferior (SI). Values were positive when deviating anteriorly, medially, or superiorly, respectively. The evaluation was done in the same fashion as previously described by Baauw et al. (2015).

Rotation was determined, clockwise values being positive and anticlockwise values being negative. In order to make rotation for left and right hips comparable, signed values were shifted for the left hips. Before measuring rotation, the difference between the planned and the postoperative anteversion and inclination was neutralized by translating the postoperative COR to the planned COR and by shifting the postoperative acetabular plane to the planned acetabular plane.

### Statistics

Due to the small number of patients descriptive statistics only was used as number of occurrences, median, and interquartile range (IQR) (Tables 2 and 3).

**Table 3.** The presented values are the average of the 2 repeated measurements (M1, M2) and show the difference between planned and postop position as well as the change in position postoperatively to 1 year follow-up. At the bottom rows, the median signed values show the direction of migration, whereas the unsigned median values show the amplitude of the migration

	Inclination (°)					Anteversion (°)				
Case	Planned	Postop.	1 year	Δ planned to postop.	Δ postop. to 1 year	Planned	Postop.	1 year	Δ planned to postop.	Δ postop.to 1 year
1	40	43.66	43.67	3.66	0.02	20	21.39	21.29	1.39	–0.11
2	40	43.60	43.66	3.60	0.06	20	25.69	25.74	5.69	0.05
3	40	51.20	51.16	11.20	–0.04	20	12.34	11.99	–7.66	–0.35
4	40	45.50	45.76	5.50	0.25	20	20.61	20.56	0.61	–0.05
5	40	45.27	45.36	5.27	0.09	20	11.98	11.84	–8.03	–0.14
6	40	41.04	40.97	1.04	–0.06	20	16.89	16.82	–3.12	–0.07
7	40	39.76	40.74	–0.24	0.98	20	7.90	7.88	–12.10	–0.02
8	40	40.99	40.92	0.98	–0.07	20	17.48	17.07	–2.53	–0.41
9	40	47.32	47.10	7.32	–0.23	20	12.95	12.70	–7.05	–0.25
10	40	29.32	29.38	–10.69	0.07	20	22.68	22.52	2.68	–0.16
Signed median				3.63	0.04				–2.82	–0.12
Unsigned median				4.46	0.07				4.40	0.12

In the bottom rows, the median signed values show the direction of deviation/migration, whereas the unsigned median values show the amplitude of deviation/migration.

Δ: difference.

### Ethics, funding, and potential conflicts of interest

The surgical option and follow-up routine for this patient category were according to the departmental norm. Thus, for this observational study, no Ethical Board Review Committee approval was necessary. However, all patients gave informed written consent to participate in the study and the follow-up examinations.

Reduced implant cost was provided for the study by the manufacturing company Materialise NV, Leuven, Belgium. GF has, in the past 3 years, given paid presentations for the same company.

## Results

There was a median deviation in postoperative position versus planned in inclination of 3.6° (IQR 1.0–5.4), in anteversion of –2.8° (IQR –7.5 to 1.2), and in rotation of –1.2° (IQR –3.3 to 0.0). The median deviation in position of COR was –0.5 mm (IQR –2.9 to 0.7) in the AP plane, –0.6 mm (IQR –1.8 to –0.1) in the ML plane, and 1.1 mm (IQR –1.6 to 2.8) in the SI plane. The migration between postoperative and 1-year follow-up caused a mean change in inclination of 0.04° (IQR –0.06 to 0.09), in anteversion of –0.13° (IQR –0.23 to –0.06), and in rotation of 0.05° (IQR –0.07 to 0.36). The migration of COR was –0.08 mm (IQR –0.18 to –0.04) in the AP plane, 0.14 mm (IQR –0.08 to 0.22) in the ML plane, and 0.06 mm (IQR –0.02 to 0.35) in the SI plane (Tables 2 and 3).

Precision analysis under repeatability conditions, assuming no bias (M1, M2), was calculated as presented in Table 2. Thus, all postoperative migration values are below the repeatability limit.

No re-revision, dislocation, infection, or fracture occurred within the 1-year follow-up. To date, none of the patients included in the study has been reoperated. 1 patient has died 2 years after surgery due to reasons not related to the hip surgery.

## Discussion

Our results are in accordance with a recently published systematic review (Chiarlone et al. 2020), indicating good short-term results and suggesting a reliable treatment option with a promising future for the treatment of severe acetabular defects. The use of this novel CT-based methodology makes it possible to create a custom-made implant that seems to provide primary implant stability, a prerequisite for satisfactory results after revision hip arthroplasty. Not only does the patient-specific implant match the acetabular defect, but its flanges outline better the ilium, ischium, and pubic bone as well. There is, however, no single surgical technique to solve the problem of cup fixation, as this is challenged by the severity of different acetabular defects. The use of impaction bone grafting (IBG) with direct cemented fixation, sometimes with metal augments or with a reinforcement cage, is considered by many to be a solid, biological fixation option in the reconstruction of large acetabular defects (Slooff et al. 1993, Sheth et al. 2013, Abolghasemian et al. 2014, Gilbody et al. 2014). Nonetheless, for these large acetabular defects, the use of IBG with metal mesh has been questioned (Buttaro et al. 2008). Other alternative fixation options include uncemented, often screw-stabilized, cups with or without bone graft or metal augments (Templeton et al. 2001, Weeden and Schmidt 2007, Macheras et al. 2009, Del Gaizo et al. 2012, Whitehouse et al. 2015). Mid- and long-term results in contained defects treated with IBG are favorable (van Egmond et al. 2011), while in segmental defects of the roof or pelvic discontinuity failures have been reported (Bonnomet et al. 2001, van Haaren et al. 2007). Desirable prerequisites for successful and durable revision include viable host bone, adequate surgical technique, and a stable and durable implant.

Reconstruction of large acetabular defects with trabecular augments or reconstruction cages can be done with well-known methods (Lopez et al. 2018, Theil et al. 2019). However, the difficulty in treating Paprosky type III acetabular defects lies in the presence of extensive bone loss, jeopardizing proper placement of acetabular components due to loss of normal bony landmarks, affecting both primary stable fixation and the restoration of the hip center. The literature shows the difficulty of accurate acetabular implant positioning (Choi et al. 2013, Citak et al. 2018) as well as a high complication rate (DeBoer et al. 2007, Taunton et al. 2012, Wind et al. 2013, Myncke et al. 2017).

We found good agreement between planned and achieved implant position regarding both rotational and translational values. During the last decades, attempts have been made to identify whether, and to what extent, early prosthetic micromotion results in later aseptic loosening (Karrholm et al. 1994). Based on the initial radiostereometric analysis, stem and socket migration exceeding 1.2 mm for the stem and 1.29 mm for the socket during the first 2 years increases the probability of revision (Karrholm et al. 1994, Nieuwenhuijse et al. 2012, Pijls et al. 2012). Although a 1-to-1 relationship between initial migration and long-term survivorship can only be assessed by long-term studies, 1-year migration values of our study are small, indicating a stable construct. The preoperative CT scanning results in a patient-specific implant bridging the acetabular defect and offers a better possibility to plan for more exact anatomical restoration. The anatomical fit of the implant, together with the pre-defined screws aiming for areas of good bone quality, minimizes possible surgical application difficulties. Altogether, the well-planned and precise process of implant positioning appears to result in a stable construct 1 year after complex acetabular revision.

At the CT1 follow-up when measuring repeatability between M1 and M2, a rather high measuring error for anteversion on the postoperative CT was explained by 1 outlier of 1.6°. ­Otherwise we consider the measurements to be reliable, as indicated in Table 2. Results are comparable to previously published studies with a similar type of concept and a very good survival rate (Colen et al. 2013, Baauw et al. 2017, Myncke et al. 2017, Goriainov et al. 2018). In a study by Citak et al. (2018), however, 1 of 9 patients suffered an implant-associated complication, after 13 months, which required revision and other complications occurred in 5 patients. A possible explanation for the complications might be that their series included even more severe cases, the revised hip had bilateral pelvic discontinuity.

A study involving 16 patients who underwent revision surgery with an associated Paprosky type III defect and a similar custom-made implant, comparing planned versus postoperative implant position with CT, indicated promising results (Baauw et al. 2015). In their study, the custom-made implant used was either a monobloc with integrated augment or in two parts as a modular construct (14 monoblocs; 2 modular constructs). Compared with our study, a higher degree of deviation from planned to postoperative position is reported, possibly related to several outliers, which may be a result of more severe bone defects. Our study is limited by the small sample size and the short follow-up time. However, all patients were included in the 1-year follow-up and no severe per- or postoperative complications were registered. To our knowledge, no previous migration study has been conducted with CT comparison between the postoperative and 1-year implant position for this type of implants.

In conclusion, our study shows good agreement between pre-planned and achieved implant position as well as a very stable construct at 1-year follow-up for these complex acetabular revision cases. This encourages us to continue the use of 3D-printed custom-made acetabular implants, including long-term follow-ups.

VZ: conduct of study, data analysis, writing of the manuscript. GF: study design and conduct, performing the surgery, data analysis, critical revision of the manuscript.

Thanks are offered to Wouter Houthoofd (Materialise NV) for technical help in performing the CT analysis and also for review of the manuscript for accuracy of the technical details, but without any influence on the interpretation and conclusion of the results. Additionally, Helene Jacobsson at Clinical Studies Sweden—Forum South, Skane University Hospital, Lund is thanked for statistical guidance.

*Acta* thanks Bart L Kaptein and Marieke Scharff-Baauw for help with peer review of this study.
